# Preparation of Water-Soluble Polyion Complex (PIC) Micelles Covered with Amphoteric Random Copolymer Shells with Pendant Sulfonate and Quaternary Amino Groups

**DOI:** 10.3390/polym10020205

**Published:** 2018-02-19

**Authors:** Rina Nakahata, Shin-ichi Yusa

**Affiliations:** Department of Applied Chemistry, University of Hyogo, 2167 Shosha, Himeji, Hyogo 671-2280, Japan; nkht9999@gmail.com

**Keywords:** amphoteric random copolymer, polyelectrolyte, polyion complex, block copolymer, polymer micelle, electrostatic interaction, protein antifouling

## Abstract

An amphoteric random copolymer (P(SA)_91_) composed of anionic sodium 2-acrylamido-2-methylpropanesulfonate (AMPS, S) and cationic 3-acrylamidopropyl trimethylammonium chloride (APTAC, A) was prepared via reversible addition-fragmentation chain transfer (RAFT) radical polymerization. The subscripts in the abbreviations indicate the degree of polymerization (DP). Furthermore, AMPS and APTAC were polymerized using a P(SA)_91_ macro-chain transfer agent to prepare an anionic diblock copolymer (P(SA)_91_S_67_) and a cationic diblock copolymer (P(SA)_91_A_88_), respectively. The DP was estimated from quantitative ^13^C NMR measurements. A stoichiometrically charge neutralized mixture of the aqueous P(SA)_91_S_67_ and P(SA)_91_A_88_ formed water-soluble polyion complex (PIC) micelles comprising PIC cores and amphoteric random copolymer shells. The PIC micelles were in a dynamic equilibrium state between PIC micelles and charge neutralized small aggregates composed of a P(SA)_91_S_67_/P(SA)_91_A_88_ pair. Interactions between PIC micelles and fetal bovine serum (FBS) in phosphate buffered saline (PBS) were evaluated by changing the hydrodynamic radius (*R*_h_) and light scattering intensity (LSI). Increases in *R*_h_ and LSI were not observed for the mixture of PIC micelles and FBS in PBS for one day. This observation suggests that there is no interaction between PIC micelles and proteins, because the PIC micelle surfaces were covered with amphoteric random copolymer shells. However, with increasing time, the diblock copolymer chains that were dissociated from PIC micelles interacted with proteins.

## 1. Introduction

A mixture of oppositely charged polyelectrolytes in water forms a water-insoluble polyion complex (PIC) due to attractive electrostatic interactions between the polymer chains [1]. Many researchers study polymer aggregates formed by electrostatic interactions. When oppositely charged diblock copolymers containing nonionic water-soluble poly(ethylene glycol) (PEG) and polyelectrolyte blocks are mixed in water, the polymers spontaneously form water-soluble PIC micelles covered with hydrophilic PEG shells [2,3]. PIC micelles can encapsulate charged compounds such as metal ions, proteins, and nucleic acid in the PIC core via electrostatic interactions [4,5,6,7,8,9,10,11]. Recently, water-soluble PIC micelles were prepared with hydrophilic poly(2-methacryloyloxyethyl phosphorylcholine) (PMPC) coronas without net charge [12,13,14]. PMPC has pendant phosphorylcholine groups with the same chemical structure as the hydrophilic part of phospholipids comprising cell membranes. The phosphorylcholine group comprises an anionic phosphonium anion and cationic quaternary amino groups. The charges in PMPC are neutralized within a single polymer chain. PMPC is a betaine polymer with good biocompatibility and antithrombogenicity [15,16,17].

Protein fouling on the surfaces of medical devices due to hydrophobic, electrostatic, and hydrogen bonding interactions causes deterioration of the functionality. Therefore, much attention has been given to the surface modification of medical devices using polymer coatings with antifouling properties. In general, protein antifouling polymers can bind water molecules strongly and are electrically neutral. When an antifouling polymer is coated on a substrate, proteins have minimal contact with the polymer on the substrate due to the presence of water molecules between the proteins and the polymers [18]. In particular, zwitterionic polymers can suppress protein adsorption, because they contain many bound water molecules [19]. Among these, betaine polymers effectively suppress protein adsorption [20,21,22]. Nanoparticles that are surface-modified with betaine polymers have an increased circulation time in the body compared to bare nanoparticles [23]. Shih et al. reported that amphoteric random copolymers with pendant anionic sulfonate and cationic quaternary amino groups inhibit protein adsorption for stoichiometrically charge neutralized compositions [24]. It is expected that amphoteric random copolymers can be applied to the surface modification of medical devices.

**Figure 1:**
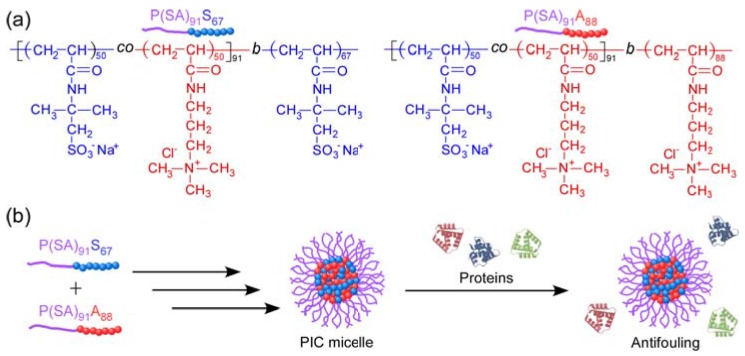
(**a**) Chemical structures of P(SA)_91_S_67_ and P(SA)_91_A_88_. (**b**) Schematic representation of water-soluble polyion complex (PIC) micelle formed from a mixture of P(SA)_91_S_67_ and P(SA)_91_A_88_; the PIC micelle shows protein antifouling properties because of its amphoteric random copolymer shell.

In the present study, we prepared an amphoteric random copolymer (P(SA)_91_) macro chain transfer agent (CTA) composed of the same amounts of anionic sodium 2-acrylamido-2-methylpropanesulfonate (AMPS, S) and cationic 3-acrylamidopropyl trimethylammonium chloride (APTAC, A) via reversible addition-fragmentation chain transfer (RAFT) radical polymerization [25]. AMPS and APTAC were polymerized using P(SA)_91_ macro-CTA to prepare an anionic diblock copolymer (P(SA)_91_S_67_) and a cationic diblock copolymer (P(SA)_91_A_88_), respectively (Figure 1). The subscripts in the abbreviations indicate the degree of polymerization (DP). Water-soluble PIC micelles were prepared by stoichiometrically charge neutralized mixing of the oppositely charged diblock copolymers, P(SA)_91_S_67_ and P(SA)_91_A_88_, in water. To the best of our knowledge, this is the first report on such micelles. The PIC micelles inhibit interactions with proteins, because their surface is covered with amphoteric random P(SA)_91_ copolymer shells.

## 2. Materials and Methods

### 2.1. Materials

2-Acrylamido-2-methylpropanesulfonic acid (AMPS, 98%) from Tokyo Chemical Industry (Tokyo, Japan) and 4,4’-azobis(4-cyanopentanoic acid) (V-501, 98%) from Wako Pure Chemical (Osaka, Japan) were used as received without further purification, and 3-acrylamidopropyl trimethylammonium chloride (APTAC, 75 wt % in water) from Tokyo Chemical Industry was passed through an Aldrich (St Louis, MO, USA) disposable inhibitor removal column. Methanol was dried with molecular sieves 3A and purified by distillation. Phosphate buffered saline (PBS) was prepared by dissolving one PBS tablet (Aldrich) in predetermined amounts of water, while 4-cyanopentanoic acid dithiobenzoate (CPD) was synthesized according to a reported method [26]. Bovine serum albumin (BSA, pH 5.0–5.6 solution) from Wako Pure Chemical and fetal bovine serum (FBS) from GE Healthcare Life Sciences HyClone were used without further purification. Water was purified using an ion-exchange column. Other reagents were used as received.

### 2.2. Preparation of P(SA)_91_

AMPS (10.0 g, 48.3 mmol) was neutralized with 6 M aqueous NaOH (10.9 mL) to adjust the pH to 6.0. APTAC (9.98 g, 48.3 mmol), V-501 (135 mg, 0.482 mmol), and CPD (270 mg, 0.965 mmol) were dissolved in a mixed solvent of MeOH (5 mL) and water (32 mL), which was added to the aqueous AMPS. The solution was degassed by purging with Ar for 30 min. Polymerization was carried out at 70 °C for 24 h. After the reaction, the total conversion of AMPS and APTAC estimated from ^1^H NMR was 90.9%. The reaction mixture was dialyzed against 1.5 M aqueous NaCl for one day, and then pure water for one day using a dialysis membrane with a molecular weight cutoff of 14 kDa (EIDIA Co. Ltd, Tokyo, Japan). The polymer (P(SA)_91_) was recovered by a freeze-drying technique (15.3 g, 71.8%). The theoretical degree of polymerization (DP(theory)) and number-average molecular weight (*M*_n_(theory)) were 91 and 1.91 × 10^4^ g/mol, respectively, estimated from the conversion and molar ratio of monomer to CPD (Formulars 1 and 2). The number-average molecular weight (*M*_n_(GPC)) and molecular weight distribution (*M*_w_/*M*_n_) were 1.54 × 10^4^ g/mol and 1.27, respectively, estimated from gel-permeation chromatography (GPC). The APTAC content was 50 mol%, estimated from quantitative ^13^C NMR measurements. The synthesis route of P(SA)_91_ is shown in Figure S1.

### 2.3. Preparation of P(SA)_91_S_67_

AMPS (771 mg, 3.72 mmol) was neutralized with 6 M aqueous NaOH (6.2 mL) to adjust the pH to 6.0. P(SA)_91_ (703 mg, 3.68 × 10^−2^ mmol, *M*_n_(theory) = 1.91 × 10^4^ g/mol, *M*_w_/*M*_n_ = 1.27) and V−501 (4.15 mg, 1.48 × 10^−2^ mmol) were added to the aqueous solution. The solution was degassed by purging with Ar for 30 min. Polymerization was carried out at 70 °C for 24 h (conversion = 90.9%). The reaction mixture was dialyzed against 1.5 M aqueous NaCl for one day, and then pure water for one day. The polymer (P(SA)_91_S_67_) was recovered by a freeze-drying technique (1.23 g, 67.6%). The degree of polymerization (DP(NMR)) of the PAMPS block was 67, estimated from quantitative ^13^C NMR measurements. *M*_n_(GPC) and *M*_w_/*M*_n_ were 2.38 × 10^4^ g/mol and 1.04, respectively, estimated from GPC. The synthesis route of P(SA)_91_S_67_ is shown in Figure S1.

### 2.4. Preparation of P(SA)_91_A_88_

APTAC (765 mg, 3.69 mmol), P(SA)_91_ (703 mg, 3.68 × 10^−2^ mmol, *M*_n_(theory) = 1.91 × 10^4^ g/mol, *M*_w_/*M*_n_ = 1.27), and V-501 (4.14 mg, 1.48 × 10^−2^ mmol) were dissolved in water (7.1 mL). The solution was degassed by purging with Ar for 30 min. Polymerization was carried out at 70 °C for 24 h (conversion = 87.7%). The reaction mixture was dialyzed against 1.5 M aqueous NaCl for one day, and then pure water for one day. The polymer (P(SA)_91_A_88_) was recovered by a freeze-drying technique (1.10 g, 74.9%). The DP(NMR) of the PAPTAC block was 88, estimated from quantitative ^13^C NMR measurements. *M*_n_(GPC) and *M*_w_/*M*_n_ were 1.87 × 10^4^ g/mol and 1.14, respectively, estimated from GPC. The synthesis route of P(SA)_91_A_88_ is shown in Figure S1.

### 2.5. Preparation of PIC micelles

P(SA)_91_S_67_ and P(SA)_91_A_88_ were dissolved separately in 0.1 M aqueous NaCl. The aqueous P(SA)_91_A_88_ was added to the P(SA)_91_S_67_ solution over a period of 5 min with stirring to prepare the PIC micelles. The mixing ratio of the polymers was represented by the molar fraction of cationic charge (*f*^+^ = [APTAC]/([AMPS] + [APTAC])). The PIC micelles were prepared at *f*^+^ = 0.5 unless otherwise noted, which represents complete charge neutralization.

### 2.6. Measurements

The ^1^H NMR and inverse-gated decoupling ^13^C NMR spectra were obtained in D_2_O using a Bruker (Yokohma, Japan) DRX-500 spectrometer. GPC measurements were performed using a Jasco (Tokyo, Japan)UV-2075 detector equipped with a Shodex (Tokyo, Japan) OHpak SB-804 HQ column working at 40 °C with a flow rate of 0.6 mL/min. An acetic acid (0.5 M) solution containing sodium sulfate (0.3 M) was used as the eluent. Sample solutions were filtered with a 0.2 µm pore size membrane filter. *M*_n_ and *M*_w_/*M*_n_ for the polymers were calibrated using standard poly(2-vinylpyridine) (P2VP) samples. Dynamic light scattering (DLS) measurements were obtained using a Malvern (Worcestershire, UK) Zetasizer Nano ZS with a He-Ne laser (4 mW at 633 nm) at 25 °C. The hydrodynamic radius (*R*_h_) was calculated using the Stokes-Einstein equation, *R*_h_ = *k*_B_*T*/(6πη*D*), where *k*_B_ is the Boltzmann constant, *T* is the absolute temperature, and η is the solvent viscosity. The DLS data was analyzed using Malvern Zetasizer software version 7.11. The angular dependence of DLS and static light scattering (SLS) was measured using an Otsuka Electronics Photal (Osaka, Japan) DLS-7000HL light scattering spectrometer equipped with a multi-*τ* digital time correlator (ALV-5000E), at 25 °C. A He-Ne laser (10 mW at 633 nm) was used as the light source [27,28,29]. The weight-average molecular weight (*M*_w_), z-average radius of gyration (*R*_g_), and second virial coefficient (*A*_2_) were estimated from SLS measurements [30]. Sample solutions for light scattering measurements were filtered using a membrane filter with 0.2 µm pores. The known Rayleigh ratio of toluene was used to calibrate the instrument. Plots of the refractive index increments against polymer concentration (d*n*/d*C*_p_) at 633 nm were obtained with an Otsuka Electronics Photal DRM-3000 differential refractometer at 25 °C. The zeta potential was measured using a Malvern Zetasizer Nano-ZS equipped with a He-Ne laser light source (4 mW, at 632.8 nm) at 25 °C. The zeta potential (ζ) was calculated from the electrophoretic mobility (μ) using the Smoluchowski relationship, ζ = ημ/ε (κ*a* >> 1), where *η* is the viscosity of the solvent, ε is the dielectric constant of the solvent, and κ and *a* are the Debye-Hückel parameter and particle radius, respectively [31]. TEM was performed with a JEOL (Tokyo, Japan) JEM-2100 microscope at an accelerating voltage of 200 kV. A sample for TEM was prepared by placing one drop of the aqueous solution on a copper grid coated with a thin film of Formvar. Excess water was blotted using filter paper. The sample was stained with sodium phosphotungstate and dried under vacuum for one day.

## 3. Results and Discussion

### 3.1. Preparation of P(SA)_91_S_67_ and P(SA)_91_A_88_

The monomer conversions (*p*) of AMPS and APTAC could not be determined individually from ^1^H NMR measurements, because the peaks of vinyl groups in the monomers completely overlapped around 5.5–6.5 ppm. The sum of *p* for the AMPS and APTAC monomers was estimated from the integral intensity ratio of the vinyl groups compared to the sum of AMPS and APTAC pendant methylene protons at 3.0–3.5 ppm after polymerization. The monomer reactivity ratios for AMPS and APTAC are assumed to be one, because the polymerizable functional groups had the same chemical structure, i.e., acrylamide-type. When random copolymerization was performed to prepare P(SA)_91_ with equal concentrations of AMPS ([*M*_AMPS_]_0_) and APTAC ([*M*_APTAC_]_0_), DP(theory) and *M*_n_(theory) were calculated from the following formulas:

(1) $$\mathrm{DP}(\mathrm{theory})=\frac{2{\left[M_{\mathrm{AMPS}}\right]}_{0}}{{\left[\mathrm{CTA}\right]}_{0}}\times \frac{p}{100}$$

(2) $$M_{n}(\mathrm{theory})=\mathrm{DP}(\mathrm{theory})\times {\mathrm{MW}}_{\mathrm{MAV}}+{\mathrm{MW}}_{\mathrm{CTA}}$$

where [CTA]_0_ is the initial concentration of CTA, MW_MAV_ is the average molecular weight of AMPS and APTAC, and MW_CTA_ is the molecular weight of CTA. When [*M*_AMPS_]_0_ = [*M*_APTAC_]_0_, the total monomer concentration becomes 2[*M*_AMPS_]_0_. The values of DP(theory) and *M*_n_(theory) are listed in Table 1.

**Figure 2:**
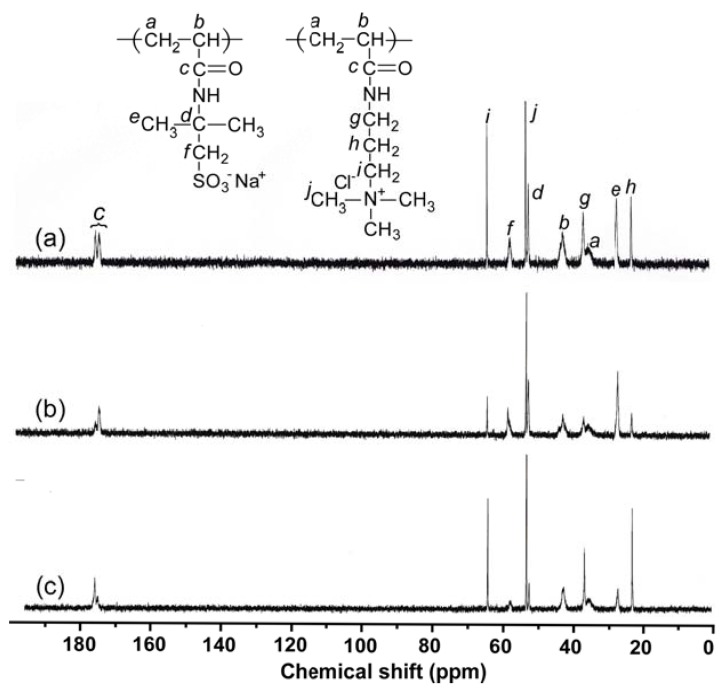
Inverse gated decoupling ^13^C NMR spectra of (**a**) P(SA)_91_, (**b**) P(SA)_91_S_67_, and (**c**) P(SA)_91_A_88_ in D_2_O.

The composition of P(SA)_91_ could not be determined from ^1^H NMR, because the peaks of the AMPS and APTAC units overlapped (Figure S2). To determine the compositions of P(SA)_91_, quantitative inverse gated decoupling ^13^C NMR measurements were performed in D_2_O (Figure 2a). The contents of AMPS and APTAC in the amphoteric random copolymer were determined from the integral intensity ratio of the peaks at 57.6 and 64.2 ppm, attributed to the pendant methylene carbons in AMPS and APTAC, respectively. The compositions of AMPS and APTAC in P(SA)_91_ were both 50 mol %. The DP(NMR) values for the PAMPS and PAPTAC blocks in P(SA)_91_S_67_ and P(SA)_91_A_88_ were found to be 67 and 88, respectively, using quantitative ^13^C NMR measurements (Figure 2b,c). The *M*_n_(NMR) values for P(SA)_91_S_67_ and P(SA)_91_A_88_ were close to the *M*_n_(theory) values. However, the *M*_n_(GPC) values for P(SA)_91_S_67_ and P(SA)_91_A_88_ deviated from the theoretical values, which indicates that there are interactions between the sample polymers and that the GPC column or the GPC standard sample (P2VP) was unsuitable for determining *M*_n_(GPC) (Figure S3). The *M*_w_/*M*_n_ values for P(SA)_91_S_67_ and P(SA)_91_A_88_ were relatively small (1.04–1.15), which suggests that the obtained polymers have well-controlled structures.

### 3.2. Preparation and Characterization of PIC Micelles

**Figure 3:**
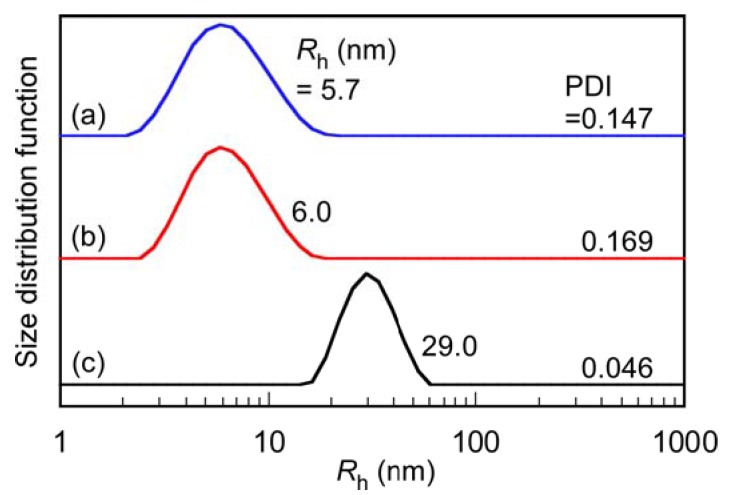
Hydrodynamic radius (*R*_h_) distributions with polydispersity indices (PDI) for (**a**) P(SA)_91_S_67_; (**b**) P(SA)_91_A_88_; and (**c**) PIC micelles in 0.1 M aqueous NaCl.

The *R*_h_ values for P(SA)_91_S_67_ and P(SA)_91_A_88_ in 0.1 M aqueous NaCl were 5.7 and 6.0 nm, respectively (Figure 3). These had unimodal distributions. The polydispersity indices (PDI) for P(SA)_91_S_67_ and P(SA)_91_A_88_ were 0.147–0.169. The *R*_h_ for the stoichiometrically charge neutralized mixture of P(SA)_91_S_67_ and P(SA)_91_A_88_ increased to 29.0 nm, which suggests the formation of PIC micelles. Assuming that the polymer main chain has a planar zig-zag structure, the expanded chain lengths of P(SA)_91_S_67_ and P(SA)_91_A_88_ are 39.5 and 44.8 nm, respectively. These are longer than the *R*_h_ of the PIC micelles (= 29.0 nm). This suggests that a PIC micelle has a simple spherical core-shell structure composed of a core formed from the anionic PAMPS and cationic PAPTAC blocks and an amphoteric P(SA)_91_ shell. The PDI for PIC micelles was narrower than for P(SA)_91_S_67_ and P(SA)_91_A_88_, which indicates that the PIC micelles have a uniform size.

The relationship between the relaxation rate (Γ) and light scattering angle (θ) was measured for PIC micelles in 0.1 M aqueous NaCl (Figure S4). The scattering vector (*q*) was calculated using *q* = (4π*n*/λ)sin(θ/2), where *n* is the refractive index of the solvent and λ is the wavelength of the light source (= 632.8 nm). The Γ-*q*^2^ plot was a straight line passing through the origin. Therefore, *R*_h_ was determined at θ = 90°, because the diffusion coefficient (*D*) was independent of θ.

**Figure 4:**
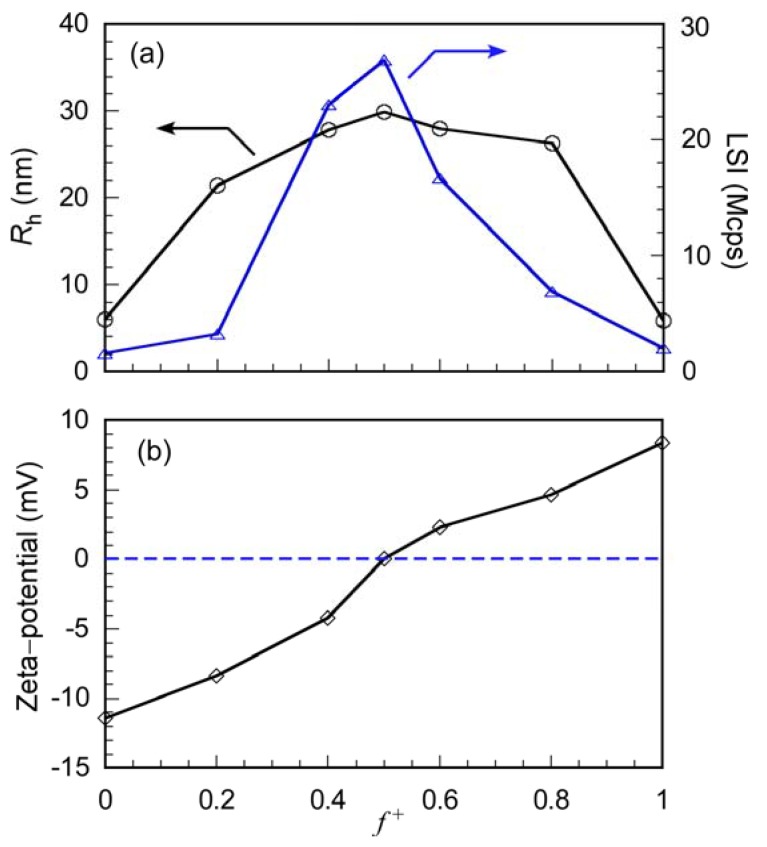
(**a**) Hydrodynamic radius (*R*_h_, ○) and light scattering intensity (LSI, △) of PIC micelles as a function of *f*^+^ (= [APTAC]/([APTAC] + [AMPS])) in 0.1 M aqueous NaCl; (**b**) Zeta potential of PIC micelles as a function of *f*^+^ in 0.1 M aqueous NaCl.

The *R*_h_, LSI, and zeta potential for the mixture of P(SA)_91_S_67_ and P(SA)_91_A_88_ at various *f*^+^ were measured in 0.1 M aqueous NaCl (Figure 4). The total polymer concentration in the solution was maintained at 1.0 g/L. We performed the LSI and zeta-potential experiments at *f*^+^ = 0 and 1 were performed at *C*_p_ = 10.0 g/L, because LSIs were too low to measure *R*_h_ and zeta-potential. The compensated LSIs at *f*^+^ = 0 and 1 are plotted in Figure 4a. At *f*^+^ = 0, the values indicated for aqueous P(SA)_91_S_67_, and the zeta potential was –11.4 mV due to the negative charge of the PAMPS block. At *f*^+^ = 1, the zeta potential for P(SA)_91_A_88_ was 8.40 mV due to the positive charge of the PAPTAC block. The zeta potential of the stoichiometrically charge neutralized mixture of P(SA)_91_S_67_ and P(SA)_91_A_88_ at *f*^+^ = 0.5 was close to 0 mV. At *f*^+^ = 0.5, the *R*_h_ and LSI had the highest values for PIC micelles. We will discuss the structure of PIC micelles in more detail, together with the results of the DLS, SLS, and TEM measurements, later in this paper. 

The *R*_h_ and LSI of PC micelles were plotted against *C*_p_ (Figure S5). *R*_h_ remained almost constant at about 30 nm and was independent of *C*_p_ in the range 0.02 ≤ *C*_p_ ≤ 1.0 g/L. LSI increased linearly with *C*_p_. These observations suggest that the shape and aggregation number (*N*_agg_), which is the number of polymer chains that form one PIC micelle, may be constant and independent of *C*_p_ in this region (0.02–1.0 g/L). At *C*_p_ ≤ 0.02 g/L, the LSI for PIC micelles was too low to obtain *R*_h_. We studied the time dependence on *R*_h_ and LSI for PIC micelles. The *R*_h_ and LSI values remained constant until at least 41 h (Figure S6).

SLS measurements for P(SA)_91_S_67_, P(SA)_91_A_88_, and PIC micelles were performed in 0.1 M aqueous NaCl (Table 2). *M*_w_ was estimated by extrapolating *C*_p_ and θ to zero in Zimm plots (Figure S7). *R*_g_ was estimated from the slope of *θ* at *C*_p_ → 0. *A*_2_ was estimated from the slope of *C*_p_ at θ → 0. The *N*_agg_ of the PIC micelles was 218, as calculated from the *M*_w_ ratio of the PIC micelles and the unimer states of P(SA)_91_S_67_ and P(SA)_91_A_88_. The *M*_w_ values of the unimer states of the block copolymers estimated from SLS were close to the weight-average molecular weight calculated from *M*_n_(NMR) and *M*_w_/*M*_n_ (Table 1). *R*_g_/*R*_h_ is useful for determining the shape of molecular aggregates. The theoretical value of *R*_g_/*R*_h_ for a homogeneous hard sphere is 0.778, while that of a random coil is about 1, and this increases substantially for a structure with a lower density and polydispersity, e.g., *R*_g_/*R*_h_ = 1.5–1.7 for flexible linear chains, and *R*_g_/*R*_h_ ≥ 2 for a rod [32]. The PIC micelles may be spherical, because *R*_g_/*R*_h_ = 0.91, which is close to 1. The *R*_g_/*R*_h_ ratios for P(SA)_91_S_67_ and P(SA)_91_A_88_ were above 1, which suggests that the block copolymer chains were relatively expanded due to electrostatic repulsions in the polyelectrolyte blocks in 0.1 M aqueous NaCl. In general, *A*_2_ relates to the interactions between a polymer chain and solvent. A large value of *A*_2_ indicates a good solvent; however, a small or negative value of *A*_2_ indicates a poor solvent [33,34]. For the PIC micelles, *A*_2_ was 1.27 × 10^−6^ cm^3^·mol/g, which was less than the corresponding values for P(SA)_91_S_67_ and P(SA)_91_A_88_ (3.73 × 10^−4^ and 3.77 × 10^−4^ cm^3^·mol/g). This indicates that the solubility of PIC micelles in 0.1 M NaCl decreased compared with those of the unimer states of the block copolymers because of the insoluble PIC core in the PIC micelles. The density (*d*) for P(SA)_91_S_67_, P(SA)_91_A_88_, and PIC micelles was calculated using the following formula:

(2) $$d=\frac{M_{w}}{N_{A}\times V}$$

where *V* is the volume of the block copolymers or PIC micelles calculated from 4/3π*R*_h_^3^. The *d* values for P(SA)_91_S_67_ and P(SA)_91_A_88_ were 0.116 and 0.0914 g/cm^3^, respectively, while *d* was 0.122 g/cm^3^ for the PIC micelles, which was slightly larger than for the diblock copolymers in the unimer states. This suggests that the polymer chains in the PIC micelles were more densely packed than those in the unimer states, because the PIC micelle core was formed by strong electrostatic interactions.

**Figure 5:**
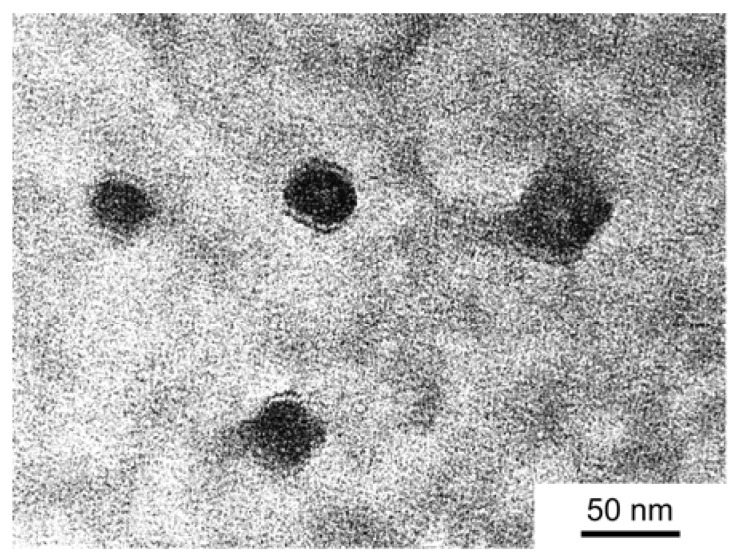
TEM image of PIC micelles with *f*^+^ = 0.5 at *C*_p_ = 1.0 g/L in 0.1 M aqueous NaCl.

TEM was performed on PIC micelles in 0.1 M aqueous NaCl (Figure 5). The average radius of a PIC micelle was 20.3 nm, estimated from TEM, which was smaller than *R*_h_ (= 29.0 nm) estimated from DLS, because the TEM sample was in the dry state. Recently, we have reported the solution properties of amphoteric random copolymers in aqueous solutions [25]. We found out that there are no interpolymer interactions of amphoteric random copolymers in 0.1 M NaCl aqueous solutions. Also, we confirmed that there are on interactions between P(SA)_91_ and anionic and cationic homopolymers using DLS measurements. From these findings, there are no interactions between amphoteric random copolymer shells in PIC micelles. Moreover, there are no interactions between amphoteric random copolymer shells and the PIC core. The PIC micelle system has the same chemical structure in the core and corona, as both domains have the same composition [35].

**Figure 6:**
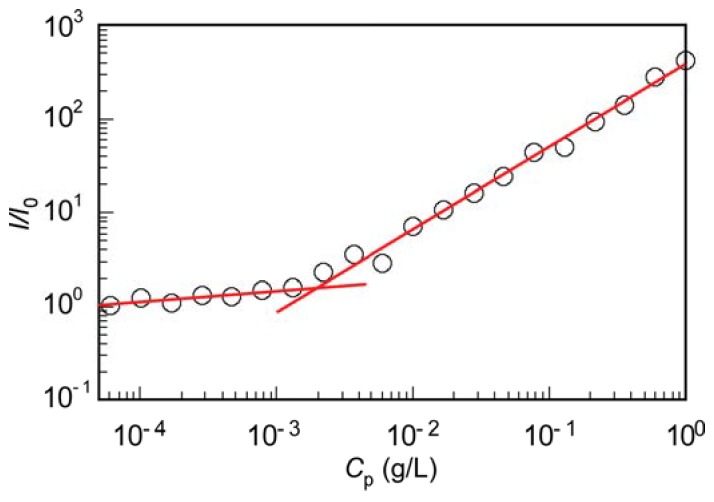
Light scattering intensity ratio (*I*/*I*_0_) for the aqueous PIC micelles containing 0.1 M NaCl as a function of polymer concentration (*C*_p_). *I* and *I*_0_ are the light scattering intensities of the solution and solvent, respectively.

The critical micelle concentration (cmc) for PIC micelles in 0.1 M aqueous NaCl was determined from the relationship between the LSI ratio (*I*/*I*_0_) and *C*_p_ (Figure 6). *I* and *I*_0_ are the LSIs of the solution and solvent, respectively. Although *R*_h_ for the PIC micelles cannot be measured for *C*_p_ ≤ 0.02 g/L, *I*/*I*_0_ can be measured below 0.02 g/L. The crossing point of the linear portions in the low and high *C*_p_ regions was 0.002 g/L, as the cmc. Below the cmc, PIC micelles may dissociate into charge neutralized small aggregates composed of a P(SA)_91_S_67_/P(SA)_91_A_88_ pair [36]. PIC micelles thus have a cmc, suggesting that they are in a dynamic equilibrium state.

**Figure 7:**
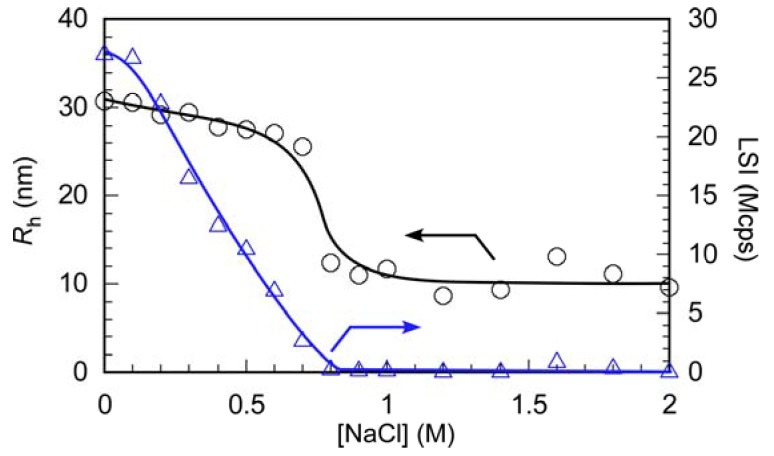
Hydrodynamic radius (*R*_h_, ○) and light scattering intensity (LSI, △) of PIC micelles with *f*^+^ = 0.5 at *C*_p_ = 1.0 g/L as a function of NaCl concentration ([NaCl]).

PIC micelles formed due to electrostatic interactions of the anionic and cationic blocks. When a low molecular weight salt such as NaCl is added to aqueous PIC micelles, their shape may change because of a screening effect [37,38]. *R*_h_ and LSI for the aqueous PIC micelles were plotted as a function of the NaCl concentration ([NaCl]) (Figure 7). At [NaCl] ≤ 0.7 M, *R*_h_ gradually decreased with increasing [NaCl], and *R*_h_ was 25.8 nm at [NaCl] = 0.7 M. *R*_h_ decreased rapidly at 0.7 M < [NaCl] ≤ 0.8 M, and *R*_h_ was about 12 nm at [NaCl] = 0.8 M. The LSI decreased almost monotonously with increasing [NaCl] until [NaCl] = 0.8 M. At [NaCl] > 0.8 M, the LSI was almost constant and independent of [NaCl]. This observation indicates that the PIC micelles partially dissociated due to the screening effect of NaCl. *R*_h_ was almost constant at 9–12 nm at [NaCl] > 0.8 M. The *R*_h_ values for the unimer states of P(SA)_91_S_67_ and P(SA)_91_A_88_ were 5.7–6.0 nm (Figure 3). The *R*_h_ values for PIC micelles at [NaCl] > 0.8 M were larger than those for the unimer states. These observations suggest that PIC micelles cannot dissociate into their unimer states at [NaCl] > 0.8 M, presumably because of salting out effects.

### 3.3. Interactions between PIC Micelles and Proteins

**Figure 8:**
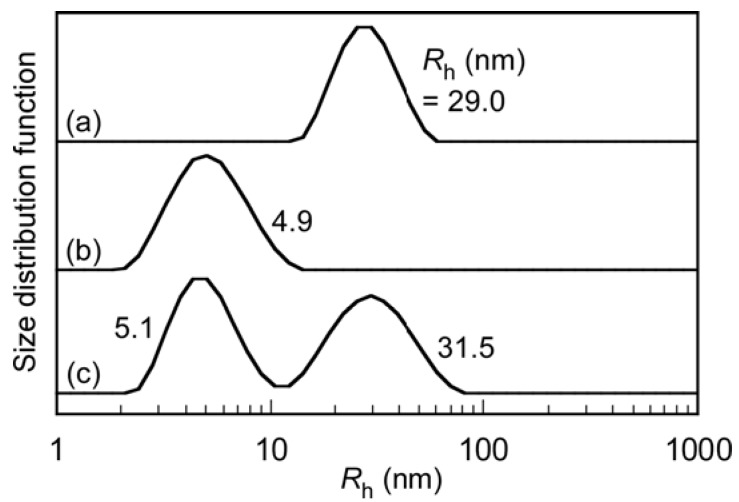
*R*_h_ distributions of (**a**) PIC micelles; (**b**) BSA; and (**c**) a mixture of PIC micelles with BSA in PBS at 25 °C.

We evaluated the interactions between PIC micelles (0.1 g/L) and BSA (5.0 g/L) in PBS using DLS (Figure 8). Before mixing, the *R*_h_ distributions were unimodal, and the *R*_h_ values for PIC micelles and BSA were 29.0 and 4.9 nm, respectively. A mixed solution of PIC micelles and BSA was prepared in PBS, and then DLS was performed on the solution within 1 h. After mixing, the distribution became bimodal, with *R*_h_ = 5.1 and 31.5 nm. The *R*_h_ value of the large size distribution was close to the *R*_h_ (= 29.0 nm) of the PIC micelles. Before mixing, the LSI values for the PIC micelles and BSA were 1.9 and 2.0 Mcps, respectively. The LSI for the mixture of PIC micelles and BSA was 2.1 Mcps, which was close to the values before mixing. This indicates that the PIC micelles do not interact considerably with BSA.

The time dependences of *R*_h_ and LSI for the mixture of PIC micelles and BSA were determined (Figure S8). The *R*_h_ distributions and LSI remained almost constant for one day. After two days, the distribution became trimodal. A small third distribution peak with an *R*_h_ of about 200 nm was observed after two days. The large aggregates with *R*_h_ ≥ 200 nm may be formed from BSA and unit PIC of a pair of P(SA)_91_S_67_/P(SA)_91_A_88_ dissociated from the PIC micelles. After four days, the area of the third distribution peak increased, and *R*_h_ increased to 281 nm. The average *R*_h_ and LSI for the mixture were almost constant for three days; however, these increased after four days. These observations suggest that the interaction between PIC micelles and proteins increased with time. The block copolymer chains can dissociate from the PIC micelles because these are in a dynamic equilibrium state. The unit PICs of P(SA)_91_S_67_/P(SA)_91_A_88_ detached from the PIC micelles may interact with proteins due to electrostatic attractions. The total charges in the unit PIC are compensated. However, dangling charge loops may be formed in the complex of cationic PAMPTAC and anionic PAMSP blocks in the unit PIC (Figure S9). In the case of PIC micelles, the surface of the PIC core was completely covered with amphoteric random copolymer shells which have protein antifouling properties. On the other hand, in the case of the unit PIC, the dangling charge loops may be exposed. The dangling charge groups in the unit PIC strongly interacted with proteins to form large aggregates.

**Figure 9:**
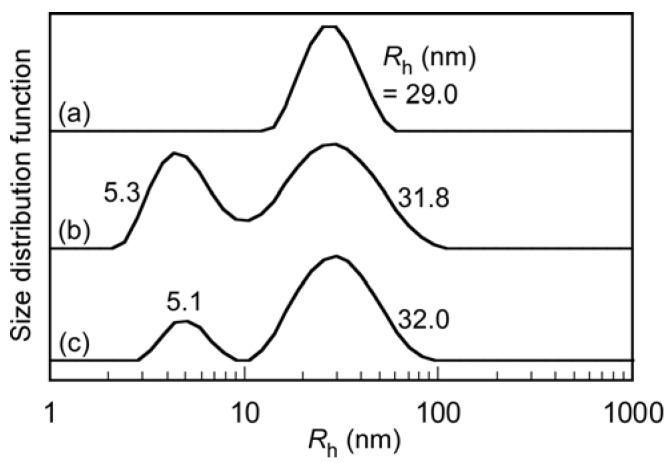
(a) *R*_h_ distributions for (**a**) PIC micelles, (**b**) FBS, and (**c**) a mixture of PIC micelles with FBS in PBS buffer at 25 °C.

The interactions between PIC micelles (0.1 g/L) and FBS (40 g/L) in PBS were evaluated by DLS (Figure 9). While BSA is a negatively charged protein, FBS is a mixture of negatively and positively charged proteins. The *R*_h_ distribution for FBS was bimodal with *R*_h_ = 5.3 and 31.8 nm. Mixed PBS solutions of PIC micelles and FBS were prepared, and then DLS was performed within 1 h. The *R*_h_ values for the mixture of PIC micelles and FBS were similar to those for FBS. The LSI of the mixed solution was 2.1 Mcps, which was similar to the values for the PIC micelles (1.9 Mcps) and FBS (2.1 Mcps) before mixing. This indicates that there is no interaction between PIC micelles and FBS. The time dependences of *R*_h_ distributions for the mixture of PIC micelles and FBS were measured (Figure S10). After two days, the distribution became trimodal. A small third distribution peak with an *R*_h_ of about 200 nm was observed after two days, which suggests that the large aggregates were formed from FBS and unit PIC formed.

## 4. Conclusions 

Oppositely charged diblock copolymers of anionic P(SA)_91_S_67_ and cationic P(SA)_91_A_88_ were prepared via RAFT using P(SA)_91_ macro-CTA. Water-soluble PIC micelles were formed from a stoichiometrically charge neutralized mixture of 0.1 M aqueous NaCl solutions of P(SA)_91_S_67_ and P(SA)_91_A_88_. The maximum values of *R*_h_ and LSI were observed when *f*^+^ was 0.5, and the zeta potential was close to 0 mV. The PIC micelles were in a dynamic equilibrium state with PIC micelles and charge neutralized small aggregates composed of a P(SA)_91_S_67_/P(SA)_91_A_88_ pair. The particle sizes in the mixture of PIC micelles and proteins in PBS remained almost constant for at least one day, which suggests that there is no interaction between PIC micelles and proteins. The PIC micelles were covered with amphoteric P(SA)_91_ shells that suppressed their interaction with proteins. However, the interactions of the diblock copolymer chains dissociated from PIC micelles and proteins increased with time. If the lengths of the PAMPS block in P(SA)_91_S_67_ and the PAPTSC block in P(SA)_91_A_88_ increased, the dynamic equilibrium may shift to form PIC micelles. Therefore, it is expected that the interaction of PIC micelles with long polyelectrolyte chains and proteins can be suppressed for a long time.

## Figures and Tables

**Table 1:** Degree of polymerization (DP), number-average molecular weight (*M*_n_), and molecular weight distribution (*M*_n_*/M*_n_).

Sample	DP (theory) *^a^*	*M*_n_(theory) *^b^* × 10^4^ (g/mol)	DP (NMR) *^c^*	*M*_n_(NMR) × 10^4^ (g/mol)	*M*_n_(GPC) × 10^4^ (g/mol)	*M*_w_/*M*_n_
P(SA)_91_	91	1.91	- *^d^*	- *^d^*	1.54	1.27
P(SA)_91_S_67_	61	3.30	67	3.27	2.38	1.04
P(SA)_91_A_88_	90	3.73	88	3.70	1.87	1.15

*^a^* Theoretical degree of polymerization estimated from Formula 1. *^b^* Theoretical number-average molecular weight estimated from Formula 2. *^c^* Estimated from quantitative inverse-gated decoupling ^13^C NMR spectra in D_2_O. *^d^* The values could not be determined from ^1^H NMR, because the terminal phenyl protons overlapped with the pendant amino protons.

**Table 2:** Dynamic light scattering (DLS) and static light scattering (SLS) data for P(SA)_91_S_67_, P(SA)_91_A_88_, and PIC micelles in 0.1 M NaCl.

Samples	M_w_ × 10^4^ (g/mol)	N_agg_	R_g_ (nm)	R_h_ (nm)	R_g_/R_h_	A_2_ × 10^−4^ (cm^3^⋅mol/g^2^)	d ^a^ (g/cm^3^)	dn/dC_p_ (mL/g)
P(SA)_91_S_67_	5.41	1	7.3	5.7	1.28	3.73	0.116	0.118
P(SA)_91_A_88_	4.98	1	6.7	6.0	1.12	3.77	0.0914	0.138
PIC micelles	752	218	26.5	29.0	0.91	0.0127	0.122	0.128

*^a^* Estimated from the values of *M*_w_ and *R*_h_ using Formula 3.

## Supplementary Materials

The following are available online at http://www.mdpi.com/2073-4360/10/2/205/s1; Figure S1: Synthesis routes of P(SA)_91_S_67_ and P(SA)_91_A_88_; Figure S2: ^1^H NMR spectra for (a) P(SA)_91_, (b) P(SA)_91_S_67_, and (c) P(SA)_91_A_88_ in D_2_O; Figure S3: GPC elution curves for (a) P(SA)_91_S_67_ and (b) P(SA)_91_A_88_ using an acetic acid (0.5 M) solution containing sodium sulfate (0.3 M) as an eluent; Figure S4: Relationship between the relaxation rate (Γ) and the square of the magnitude of the scattering intensity vector (*q*^2^) for PIC micelles at *C*_p_ = 1 g/L in 0.1 M aqueous NaCl at 25 °C; Figure S5: Hydrodynamic radius (*R*_h_, ○) and light scattering intensity (LSI, △) of PIC micelles as a function of polymer concentration (*C*_p_) in 0.1 M aqueous NaCl. Figure S6: Relationship between *R*_h_ (○) and light scattering intensity (LSI, △) as a function of time for PIC micelle with *f*^+^ = 0.5 at *C*_p_ = 1.0 g/L in 0.1 M NaCl aqueous solution; Figure S7: A typical Zimm plot for PIC micelles in 0.1 M aqueous NaCl at 25 °C; Figure S8: (a) Relationship between *R*_h_ (○) and light scattering intensity (LSI, △) as a function of time, and (b) *R*_h_ distributions for a mixture of PIC micelles/BSA at *C*_p_ = 0.1 g/L and [BSA] = 5.0 g/L in PBS at 25 °C; Figure S9: Conceptual illustration of dangling charge groups in the unit PIC of P(SA)_91_S_67_/P(SA)_91_A_88_; Figure S10: *R*_h_ distributions for mixture of PIC micelle/FBS at *C*_p_ = 0.1 g/L and [FBS] = 40 g/L in PBS at 25 °C.
