# Supplementary material for: Preparation of Water-Soluble Polyion Complex (PIC) Micelles Covered with Amphoteric Random Copolymer Shells with Pendant Sulfonate and Quaternary Amino Groups
Source: Polymers (Basel). 2018 Feb 19;10(2):205. doi: 10.3390/polym10020205 (PMC6415021; doi:10.3390/polym10020205)
Supplement: Supplementary file 1 [file polymers-10-00205-s001.pdf]

Electronic supplementary information (ESI)

Preparation of Water-soluble Polyion Complex (PIC) Micelles Covered with Amphoteric Random Copolymer Shells with Pendant Sulfonate and Quaternary Amino Groups

Rina Nakahata and Shin-ichi Yusa*

Department of Applied Chemistry, University of Hyogo, 2167 Shosha, Himeji, Hyogo 671-2280, Japan; nkht9999@gmail.com (R.N.); yusa@eng.u-hyogo.ac.jp (S.Y.)

* Correspondence: yusa@eng.u-hyogo.ac.jp; Tel.: +81-79-267-4954

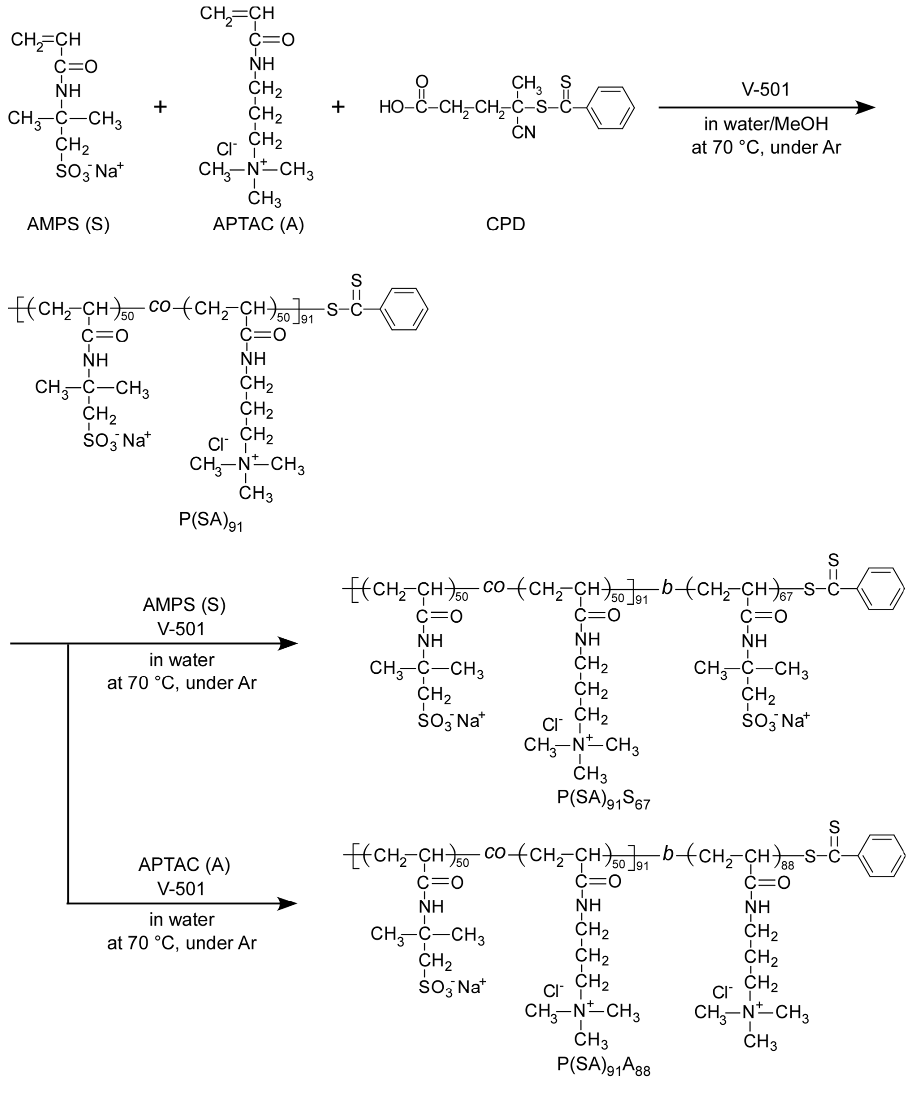

Figure S1. Synthesis routes of P(SA)$_{91}$S$_{67}$ and P(SA)$_{91}$A$_{88}$.

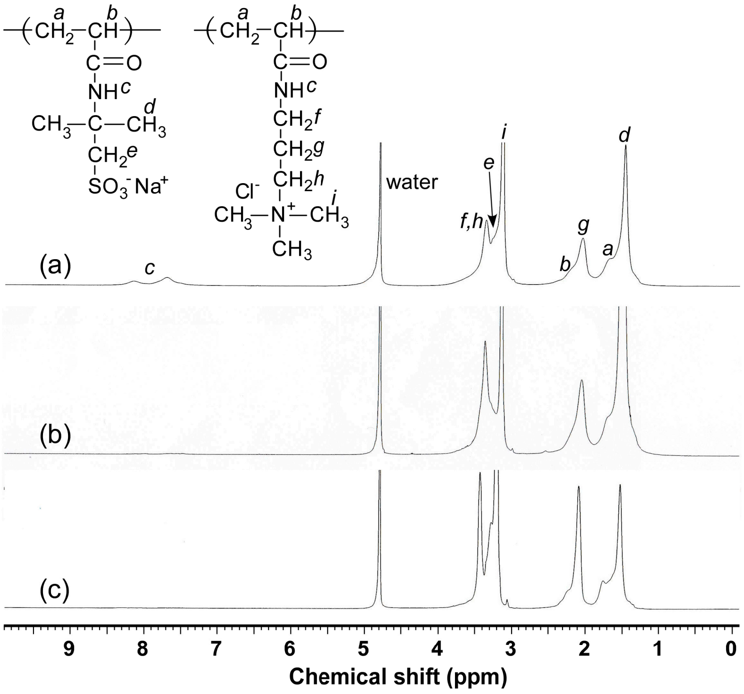

Figure S2. ^{1}H NMR spectra for (a) $P(SA)_{91}$, (b) $P(SA)_{91}S_{67}$, and (c) $P(SA)_{91}A_{88}$ in D_2O.

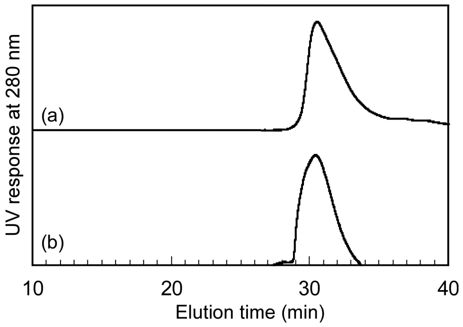

Figure S3. GPC elution curves for (a) $P(SA)_{91}S_{67}$ and (b) $P(SA)_{91}A_{88}$ using an acetic acid (0.5 M) solution containing sodium sulfate (0.3 M) as an eluent.

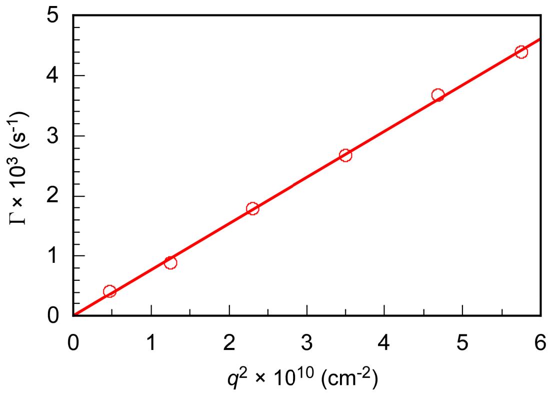

Figure S4. Relationship between the relaxation rate (Γ) and the square of the magnitude of the scattering intensity vector (q^2) for PIC micelles at C_p = 1 g/L in 0.1 M aqueous NaCl at 25 °C.

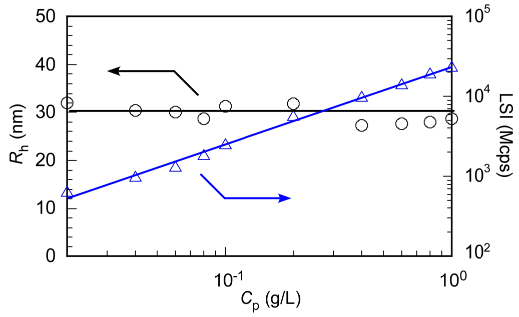

Figure S5. Hydrodynamic radius (R_h, ○) and light scattering intensity (LSI, △) of PIC micelles as a function of polymer concentration (C_p) in 0.1 M aqueous NaCl.

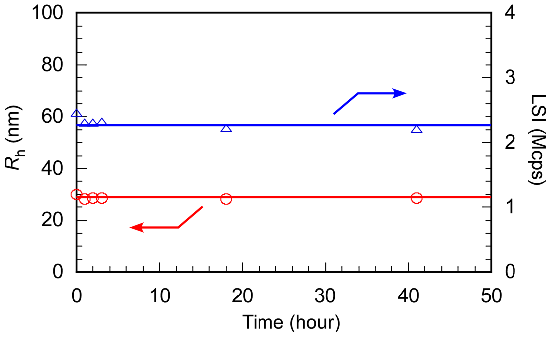

Figure 6. Relationship between R_h (○) and light scattering intensity (LSI, △) as a function of time for PIC micelle with f^+ = 0.5 at C_p = 1.0 g/L in 0.1 M NaCl aqueous solution.

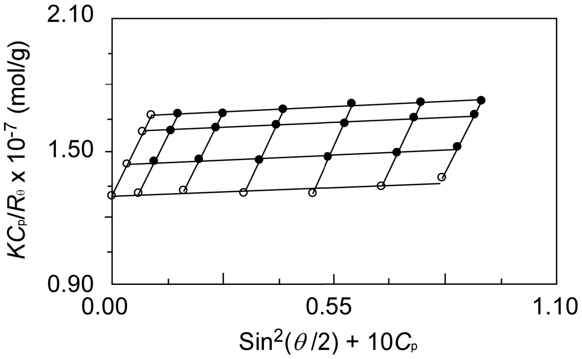

Figure S7. A typical Zimm plot for PIC micelles in 0.1 M aqueous NaCl at 25 °C.

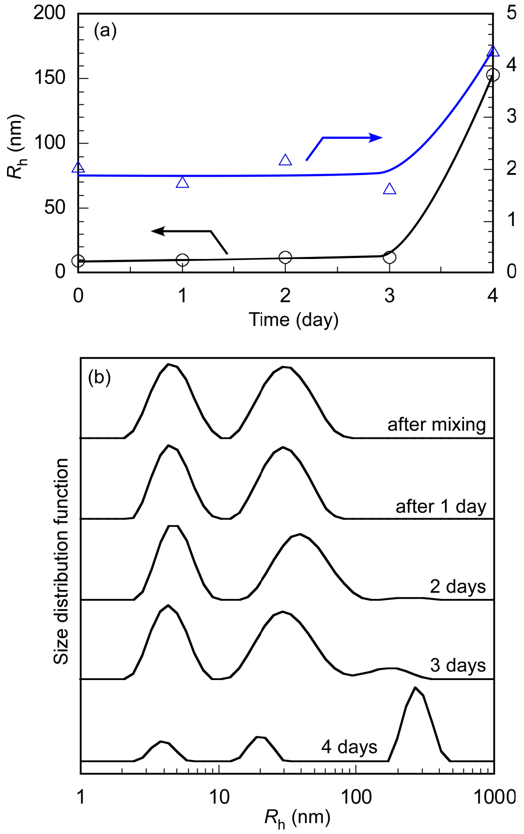

Figure S8. (a) Relationship between R_h (○) and light scattering intensity (LSI, △) as a function of time, and (b) R_h distributions for a mixture of PIC micelles/BSA at C_p = 0.1 g/L and [BSA] = 5.0 g/L in PBS at 25 °C.

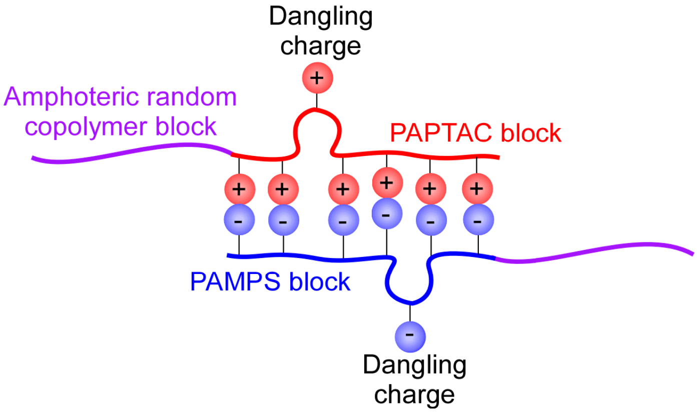

Figure S9. Conceptual illustration of dangling charge groups in the unit PIC of $P(SA)_{91}S_{67}/P(SA)_{91}A_{88}$.

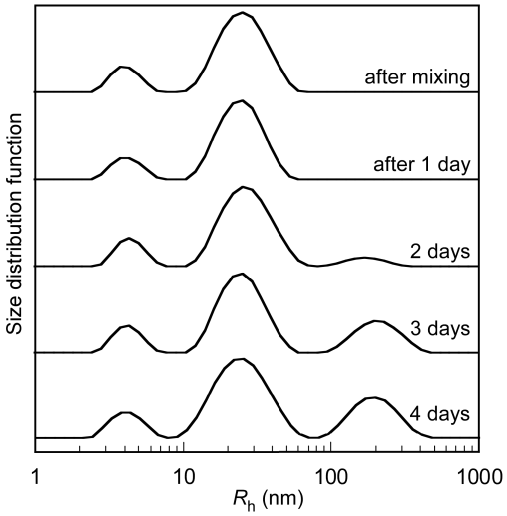

Figure S10. R_h distributions for mixture of PIC micelle/FBS at C_p = 0.1 g/L and [FBS] = 40 g/L in PBS at 25 °C.
